# The sparing effect of ultra-high dose rate irradiation on the esophagus

**DOI:** 10.3389/fonc.2024.1442627

**Published:** 2024-07-12

**Authors:** Wenting Ren, Lu Hou, Ke Zhang, Huan Chen, Xin Feng, Ziming Jiang, Fei Shao, Jianrong Dai, Yibo Gao, Jie He

**Affiliations:** ^1^ Department of Radiation Oncology, National Cancer Center/National Clinical Research Center for Cancer/Cancer Hospital, Chinese Academy of Medical Sciences and Peking Union Medical College, Beijing, China; ^2^ Department of Thoracic Surgery, National Cancer Center/National Clinical Research Center for Cancer/Cancer Hospital, Chinese Academy of Medical Sciences and Peking Union Medical College, Beijing, China; ^3^ Laboratory of Translational Medicine, National Cancer Center/National Clinical Research Center for Cancer/Cancer Hospital, Chinese Academy of Medical Sciences and Peking Union Medical College, Beijing, China; ^4^ State Key Laboratory of Molecular Oncology, National Cancer Center/National Clinical Research Center for Cancer/Cancer Hospital, Chinese Academy of Medical Sciences and Peking Union Medical College, Beijing, China; ^5^ Central Laboratory and Shenzhen Key Laboratory of Epigenetics and Precision Medicine for Cancers, National Cancer Center/National Clinical Research Center for Cancer/Cancer Hospital and Shenzhen Hospital, Chinese Academy of Medical Sciences and Peking Union Medical College, Shenzhen, China

**Keywords:** FLASH, irradiation, sparing effect, esophagus, proteomic

## Abstract

**Background and purpose:**

Current studies have substantiated the sparing effect of ultra-high dose rate irradiation (FLASH) in various organs including the brain, lungs, and intestines. Whether this sparing effect extends to esophageal tissue remains unexplored. This study aims to compare the different responses of esophageal tissue in histological and protein expression levels following conventional dose rate irradiation (CONV) and FLASH irradiation to ascertain the presence of a sparing effect.

**Methods and materials:**

C57 female mice were randomly divided into three groups: control, CONV, and FLASH groups. The chest region of the mice in the radiation groups was exposed to a prescribed dose of 20 Gy using a modified electron linear accelerator. The CONV group received an average dose rate of 0.1 Gy/s, while the FLASH group received an average dose rate of 125 Gy/s. On the 10th day after irradiation, the mice were euthanized and their esophagi were collected for histopathological analysis. Subsequently, label-free proteomic quantification analysis was performed on esophageal tissue. The validation process involved analyzing transmission electron microscopy images and utilizing the parallel reaction monitoring method.

**Results:**

Histopathology results indicated a significantly lower extent of esophageal tissue damage in the FLASH group compared to the CONV group (*p* < 0.05). Label-free quantitative proteomic analysis revealed that the sparing effect observed in the FLASH group may be attributed to a reduction in radiation-induced protein damage associated with mitochondrial functions, including proteins involved in the tricarboxylic acid cycle and oxidative phosphorylation, as well as a decrease in acute inflammatory responses.

**Conclusions:**

Compared with CONV irradiation, a sparing effect on esophageal tissue can be observed after FLASH irradiation. This sparing effect is associated with alleviated mitochondria damage and acute inflammation.

## Introduction

The global incidence of esophageal cancer ranks seventh, while its mortality ranks sixth (1), with an overall 5-year survival rate of 20% (2). Radiotherapy (RT) plays a crucial role in the comprehensive treatment of esophageal cancer, which covers preoperative neoadjuvant, postoperative adjuvant, curative, and palliative treatments (3). However, RT also has risks or side effects, which include radiation esophagitis (4), radiation pneumonitis (5), esophageal obstruction (6), or even esophageal perforation (7). The radiation-induced side effects may have a detrimental impact on the life quality of patients undergoing treatment, necessitating clinicians to strike a delicate balance between irradiation dosage and the risk of radiation-induced side effects. When RT is administered with chemotherapy and immunotherapy, the side-effect-related toxicity may aggravate, especially pulmonary toxicity (8, 9).

Recently, ultra-high dose rate irradiation, which is widely known as FLASH, has emerged as one of the most promising and debated techniques in radiotherapy (10, 11). Different from the conventional irradiation (CONV) with a mean dose rate of 0.1 Gy/s, an ultra-high dose rate over 40 Gy/s was realized by FLASH irradiation. In some preclinical or clinical studies, FLASH irradiation displayed the potential to mitigate the toxicity of non-malignant tissue surrounding the tumor while maintaining comparable therapeutic efficacy to CONV (12, 13). This mitigation in toxicity on normal tissues after FLASH irradiation is widely known as the sparing effect, which has been further confirmed in a series of studies involving neuroinflammation, pneumonia, pulmonary fibrosis, and intestinal damage (14–16). However, the degree of FLASH-mediated sparing effects varies from different tissues and organs. Presently, there is a study systematically investigating the sparing effect of FLASH irradiation on various organs in animal experiments, with CONV irradiation as the control group. In the study, a concept of FLASH-modifying factors was introduced to facilitate a more objective comparison of the degree of radiation toxicity remission in different organs after FLASH irradiation (17). The results suggested that the response varied across different organs, emphasizing the necessity to broaden the research scope of FLASH irradiation techniques for diverse tissues and organs. However, research on the potential toxicity damage of FLASH irradiation to esophageal tissue remains unexplored. It is uncertain whether employing FLASH irradiation can effectively alleviate the side effects during radiotherapy of esophageal cancer.

Meanwhile, the comprehensive biological mechanisms of FLASH irradiation remain not entirely clear (10, 18). Insights from mechanistic studies of CONV irradiation inform us that radiation response is a complex process unfolding at various cellular and molecular levels (19–21). This process includes DNA damage, organelle impairment, cell cycle arrest and apoptosis, inflammation and immune reactions, radiation-induced oxidative stress responses, and genetic and epigenetic effects (22). In terms of FLASH irradiation, published studies have reported a reduction in DNA damage (23, 24), decreased mitochondrial damage, reduced cell apoptosis (18), and alleviated inflammatory reactions among the aforementioned mechanisms (13). However, it is still unknown whether these sparing effects can be observed in esophageal tissue during FLASH irradiation, and the cellular or molecular mechanisms of FLASH sparing effect on esophageal tissue still need to be explored.

For now, this is the first study focusing on the sparing effect of FLASH irradiation on esophageal tissue. The extent of histopathological damage in the esophagus was observed in mice treated with CONV and FLASH irradiation and compared to that of the control group (non-anesthetized, non-irradiated). The protein level differences among these groups were analyzed by the high-throughput label-free proteomic quantification method, which provided a theoretical basis for revealing the mechanism of sparing effect resulting from FLASH irradiation. The protein expression responses regarding the sparing effect of FLASH irradiation on the esophagus were also validated using transmission electron microscopy (TEM) image analysis and the parallel reaction monitoring (PRM) method.

## Materials and methods

### Study design

The present study reported a radiotherapy experiment *in vivo*. The goal was to compare the histopathological responses and protein expression levels in the esophageal tissue of mice exposed to either FLASH or CONV irradiation. According to the intervention methods, three groups were established: control, CONV, and FLASH groups.

The mice were randomly divided into control, CONV, and FLASH groups based on the random numbers generated by the standard =RAND function in Microsoft Excel. Each individual mouse was considered as an experimental unit in the present study. The processes of irradiation application and dosimetry measurement were described in detail in the subsequent sections. On the 10th day after irradiation, mice in different groups were euthanized and their esophagi were harvested for subsequent histopathological and proteomic analyses. Mice with deviations from the prescribed dose (higher or lower than the designed dose) during FLASH irradiation or with premature death were excluded from sampling.

Histopathological analysis and label-free proteomic quantification analysis were used to assess the different responses in the esophageal tissue of mice exposed to FLASH or CONV irradiation. TEM and the parallel reaction monitoring method were used to validate the findings in the histopathological and label-free proteomic quantification analyses.

### Animals

The animal experiments conducted in this study were approved by the ethics committee of our institution. Twenty-seven female mice (C57 BL/6N) 8 weeks of age were procured from Beijing Vital River Laboratory Animal Technology. We opted for a small sample size in accordance with ethical guidelines while ensuring statistical robustness. All of the mice were housed in a specific pathogen-free environment, maintaining a constant temperature of 20°C–26°C, relative humidity between 40% and 70%, and a 12/12-h light/dark cycle at the animal center. The environment and housing facilities for laboratory animals adhered to the requirements outlined in the national standard GB14925–2010. After adaptive feeding, the mice were randomly divided into control, CONV irradiation, and FLASH irradiation groups (*n* = 9).

### Irradiation

Irradiation was conducted using a clinical accelerator located at Elekta Asia Pacific Center for Learning and Innovation in Beijing, China. The clinical accelerator was modified to generate ultra-high dose rate pulsed electron beams suitable for FLASH irradiations, as detailed in previously published methods (25). FLASH irradiations were executed at an instantaneous dose rate exceeding 1 × 10^6^ Gy/s within each pulse, with an average dose rate of 125 Gy/s. The conventional dose rate was 0.1 Gy/s, following the clinical treatment mode. The detailed beam and prescription dose parameters employed throughout this study are provided in **Supplementary Table S1**.

During FLASH irradiation, the gantry angle was set at 180°. The accelerator was disassembled, removing the treatment head, collimator, and filter while retaining only the cable connections. The radiation positioning device for mice was directly placed on the radiation head to achieve the maximum FLASH radiation dose rate. The positioning device consisted of a custom-built lead with a thickness of 1 cm and a 2.5-cm × 3.5-cm hollow in the middle, allowing the chest of each mouse to be in the irradiated field while the rest of the body was shielded by lead. FLASH mode irradiated one mouse at a time due to the FLASH radiation field limitation. During CONV irradiation, a lead block with a 2.5-cm × 10-cm hollow in the middle was used, allowing for the irradiation of three mice simultaneously and reducing the overall irradiation duration (**Supplementary Figure S1A**). Although the hollow sizes of the lead blocks differ between the two irradiation modes, the doses received by mice under both modes remained consistent. The flatness and symmetry of the dose distributions within each mode were confirmed using Gafchromic™ EBT-XD films (Ashland Inc., Bridgewater, NJ, USA) (**Supplementary Figure S1B**). The irradiation sequence of mice within the same group was randomized.

### Dosimetry

The dose distributions of different depths for FLASH irradiation were measured using EBT-XD film at various depths in polymethyl methacrylate with different thicknesses. From the results of the FLASH irradiation mode, there is no apparent dose buildup region (**Supplementary Figure S1D**). Therefore, CONV irradiation included an additional 1-cm-thick tissue compensator as a buildup region to ensure comparability of the depth dose within tissues between the two irradiation modes. All animal irradiations were performed under anesthesia (tribromoethanol, 250 mg/kg intraperitoneally).

Individual mouse dosimetry was conducted using EBT-XD films, which were positioned in front of the chest during irradiation. Optical density (OD) measurements of the films were obtained immediately after irradiation using a dedicated meter. By establishing a pre-existing relationship between OD value and dose, the instantaneous dose can be determined. The precise doses and dose distributions were obtained 48 h after RT through an Epson scanner (https://epson.com/scanners) and RIT software (https://radimage.com). It should be noted that occasional dose instability may occur due to the high intensity of the FLASH electron beam. Two mice in the FLASH group were excluded due to deviations from the prescribed dose during FLASH irradiation based on the results of the individual mouse dosimetry measurement.

### Histopathology

In the CONV group, two mice expired prior to esophageal tissue collection and were subsequently excluded. The remaining mice in the CONV group (*n* = 7) and FLASH group (*n* = 7) were euthanized painlessly, and the entire esophagus was collected for subsequent analysis (**Supplementary Figure S1E**). As a sham comparison, seven mice in the control group were also randomly selected for esophageal tissue collection and further analyses. The middle part of the esophagus of each mouse was gently washed with ice-cold PBS, fixed in 10% paraformaldehyde for 3 h in a cold room, embedded in paraffin, and sectioned into 7-μm-thick slices for histopathology analysis. The tissue sections were stained with hematoxylin–eosin (H&E). Pathological slides were examined by an animal pathology analyst with over 30 years of experience. The extent of damage was assessed based on histological morphology and inflammatory infiltration degree using the following four-grade categorization system referenced from existing literature (26, 27): none, the esophageal tissue was normal; +, essentially normal tissues with degeneration or loss were observed in individual cells of superficial and basal layers along with slight inflammatory cell infiltration; ++, varying degrees of degeneration and necrosis were observed in the superficial and basal layers accompanied by some inflammatory cell infiltration; and +++, complete disappearance or disintegration of original stratified squamous epithelium structure in the esophagus along with extensive inflammatory cell infiltration was observed.

### Protein extraction, digestion, and detection

The remaining esophageal tissue from each group was divided into three replicates, with two, two, and three mice mixed in each replicate. Subsequently, the tissue was ground using liquid nitrogen. Next, a lysis buffer (consisting of 1% Triton X-100, 1% protease inhibitor, 50 μM of PR-619, 3 μM of TSA, 50 mM of NAM) was added for ultrasonic cracking. The protein concentrations were determined using the BCA kit, followed by trypsin digestion to convert the proteins into peptides. These resulting peptides were then dissolved in liquid chromatography mobile phase A and separated using the EASY-nLC 1200 ultra-high-performance liquid system. The separated peptides were subsequently analyzed by Orbitrap Exploris™ 480 mass spectrometry for detection. The raw data obtained from this analysis were imported into Proteome Discoverer (v2.4.1.15) for database searches against the Mus_musculus_10090_SP_20210721.fasta database. Quality control analysis was conducted at both the peptide and protein levels based on the outcomes of these database searches.

### Bioinformatics analysis for protein characterization

The identified proteins were subjected to common functional annotations, including Gene Ontology (GO) and Kyoto Encyclopedia of Genes and Genomes (KEGG), using the eggNOG mapper software (v2.0) based on the eggNOG database (v5.0.2, http://eggnog5.embl.de/#/app/home). Additionally, the diamond software (v2.0) based on KEGG mapper (v5.0, http://www.kegg.jp/kegg/mapper.html) was employed for this purpose as well. The subcellular locations of proteins were determined using the PSORTb software (v3.0 https://www.psort.org/psortb/), while Circos v0.69–9 software was utilized for visualization. The fold change (FC) for three replicates was calculated as the ratio of the mean relative quantitative value of each protein in multiple replicates. Significantly upregulated changes were defined as those with a differential expression level change greater than 1.5 when the *p*-value <0.05, while significantly downregulated changes were defined as those less than 2/3. Differentially expressed proteins in each comparison group were respectively enriched at two levels: GO and KEGG using Fisher’s exact test. Hierarchical clustering analysis was performed to cluster related functions across different groups using the K-means algorithm. Mfuzz clustering analysis was conducted using the R package Mfuzz based on the fuzzy c-means algorithm (v.2.32.0 https://www.rdocumentation.org/packages/Mfuzz/versions/2.32.0).

### Transmission electron microscopy analysis

The electron microscopy analysis was conducted on three mice selected from each group. The esophageal tissue was promptly excised from euthanized mice within 1–3 min postmortem and fixed in an electron microscope fixative solution, followed by dehydration, infiltration, embedding, sectioning, and uranium-lead double staining (2% uranyl acetate saturated aqueous solution, lead citrate, each staining for 15 min). Tissue morphology was examined using a transmission electron microscope (TEM, Hitachi HT7800), and images were captured for subsequent analysis.

### Parallel reaction monitoring validation

PRM is an ion monitoring technology that relies on high-resolution and high-precision mass spectrometry to achieve accurate quantification of target proteins. The methods for PRM verification have been previously described in published studies (28, 29). In this study, consistent with the proteomic analysis, the PRM validation also included three replicates in each group. The experimental procedures involved protein extraction, trypsin digestion, mass spectrometry analysis, and data analysis. Tandem mass spectrometry (MS/MS) analysis was performed using Q Exactive™ Plus (Thermo), while MS data processing was conducted using Skyline (v.21.1). The enzyme was set as trypsin [KR/P] and the max missed cleavage set as 0. Peptide length was set as 7–25 amino acid residues. Finally, the target protein was visualized in a bubble diagram using the ggplot2 package (R; v.4.2.2).

### Statistical analysis

The *t*-test was employed for comparing continuous variables between two groups, while one-way ANOVA was utilized for comparisons involving more than two groups. Categorical variables were compared using Fisher’s exact test.

## Results

### FLASH irradiation reduces acute damage to esophageal tissue

**Figure 1:**
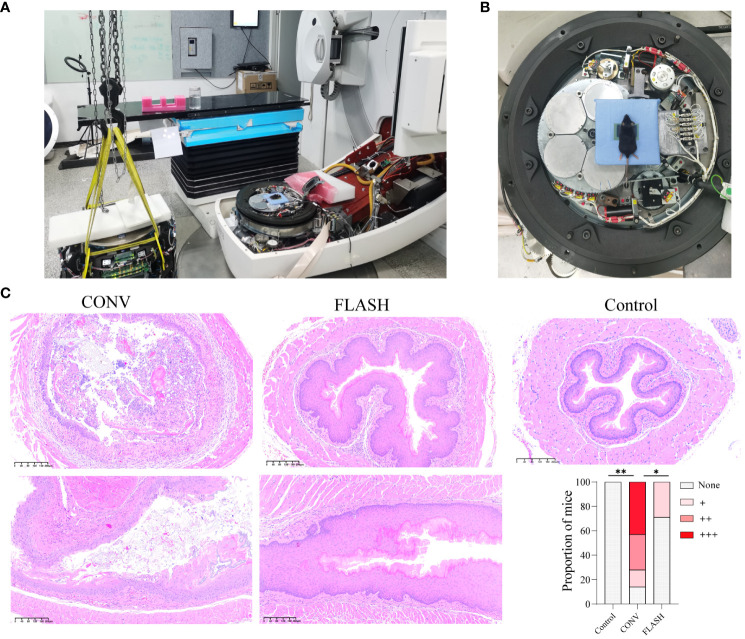
FLASH irradiation setup and histopathological analysis of esophageal tissue. **(A)** FLASH irradiation setup. The accelerator was disassembled, removing the treatment head, collimator, and filter while retaining the cable connections. **(B)** FLASH irradiation positioning. Mice were positioned on the scattering plate of the electron beam using a custom-built lead block, and the film in front of the chest was utilized to confirm the individual mouse dose. **(C)** The representative histopathological images of esophageal tissue from mice on the 10th day after initial irradiation, depicting both transversal and coronal sections. The bar chart illustrates the histopathological scores (n = 7 for each group). *: p < 0.05, **: p < 0.01.

The FLASH irradiation was delivered utilizing a modified clinical accelerator (**Figure 1A**) and achieved an average dose rate of 125 Gy/s. The FLASH irradiation was observed to generate a comparable and uniformly distributed dose throughout the irradiated volume in comparison with CONV irradiation (**Supplementary Figure S1C**). The mice were positioned directly on the accelerator’s electron beam scattering foil for irradiation (**Figure 1B**). The representative H&E images of esophageal tissue taken on the 10th day after initial irradiation, along with the corresponding bar chart of pathological scores, are illustrated in **Figure 1C**. In the CONV group, H&E staining results revealed that 43% of the mice exhibited severe cell necrosis accompanied by inflammatory infiltration in the esophageal tissue, while another 29% of mice displayed a moderate degree of degenerative necrosis and inflammation. Furthermore, 14% of the mice showed some cell degeneration or loss, accompanied by mild inflammatory infiltration in esophageal tissue. The remaining 14% of the mice displayed structurally normal esophageal tissue. In the FLASH group, only 29% of the mice were associated with some cell degeneration or loss accompanied by mild inflammatory infiltration, while the remaining 71% of the mice were essentially normal based on the H&E staining results of esophageal tissue. The pathological score grades displayed a significant difference between the CONV and control (*p* = 0.001) and the CONV and FLASH groups (*p* = 0.046).

### Proteomic profiling unravels distinct esophageal responses to FLASH and CONV irradiation

**Figure 2:**
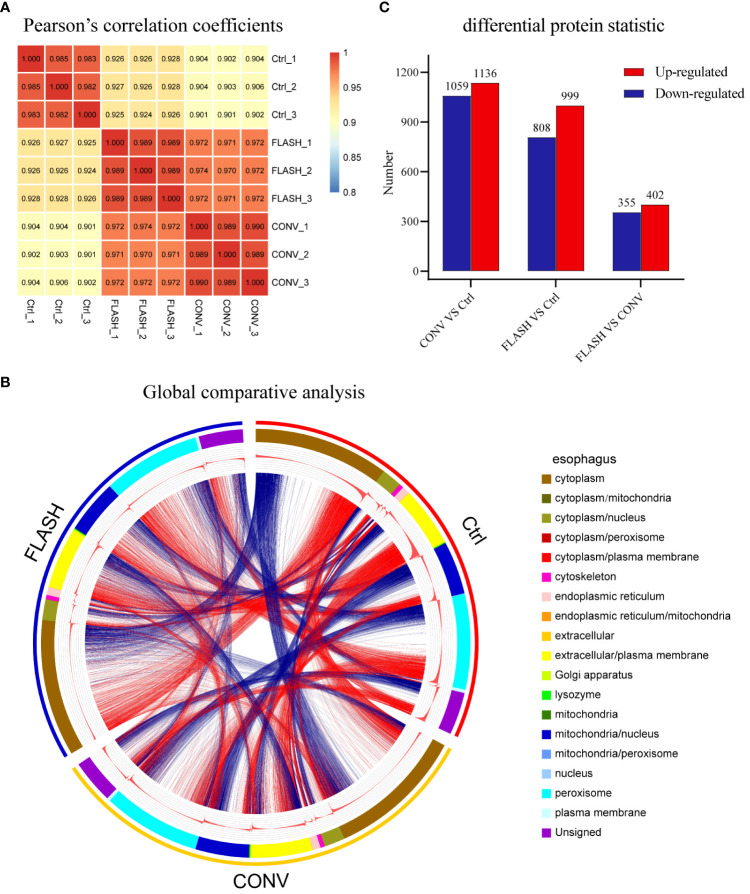
The overview results of proteomic analysis. **(A)** Heatmap of Pearson’s correlation coefficients (PCCs) among all samples. **(B)** Circos diagram (the first circle represents the sample name, the second circle represents subcellular location information of proteins, the third circle represents the expression abundance of proteins, and the fourth circle represents the differences among different groups). **(C)** Overall map of differentially expressed upregulated and downregulated proteins in each comparison group (*n* = 3 for each group).

A total of 5,669 proteins were identified and 4,851 proteins were quantified (**Supplementary Figure S2A**). Pearson’s correlation coefficient (PCC) analysis and principal component analysis (PCA) revealed outstanding consistency among the three replicates within each group (**Figure 2A**; **Supplementary Figure S2B**), ensuring the assessment of genuine biological relevance. A global comparative analysis of esophagus tissue among various groups was subsequently performed to investigate the entire proteomic changes. We used a Circos diagram to display the results of the collection of protein locations and expression abundances for samples after different treatments (**Figure 2B**). The first circle is labeled by the group names to signify that the sample underwent different treatments. The second circle represents subcellular location information of proteins, while their corresponding expression abundance is depicted in the third circle. The fourth circle comprises the same proteins exhibiting expression differences across various treatment groups. By connecting the lines and the number of lines, it was demonstrated that significant changes in the expression levels of multiple proteins within various subcellular structures occurred following irradiation. Intriguingly, there were also numerous connecting lines between the FLASH and CONV groups, indicating a distinct protein expression difference induced by FLASH and CONV irradiation (**Figure 2B**). Then, the significant number of dysregulated proteins after irradiation was identified based on proteomic data, with |log2 (fold change) | >1.5 and *p*-value <0.05 as the screening criteria. Compared with the control group, 1,136 upregulated differentially expressed proteins (DEPs) and 1,059 downregulated DEPs were obtained after CONV irradiation, while 999 upregulated DEPs and 808 downregulated DEPs were obtained after FLASH irradiation. When comparing FLASH irradiation to CONV irradiation, 402 upregulated DEPs and 355 downregulated DEPs were obtained (**Figure 2C**).

### Cluster analysis reveals the biological processes and pathways with the greatest differences between the FLASH and CONV groups

**Figure 3:**
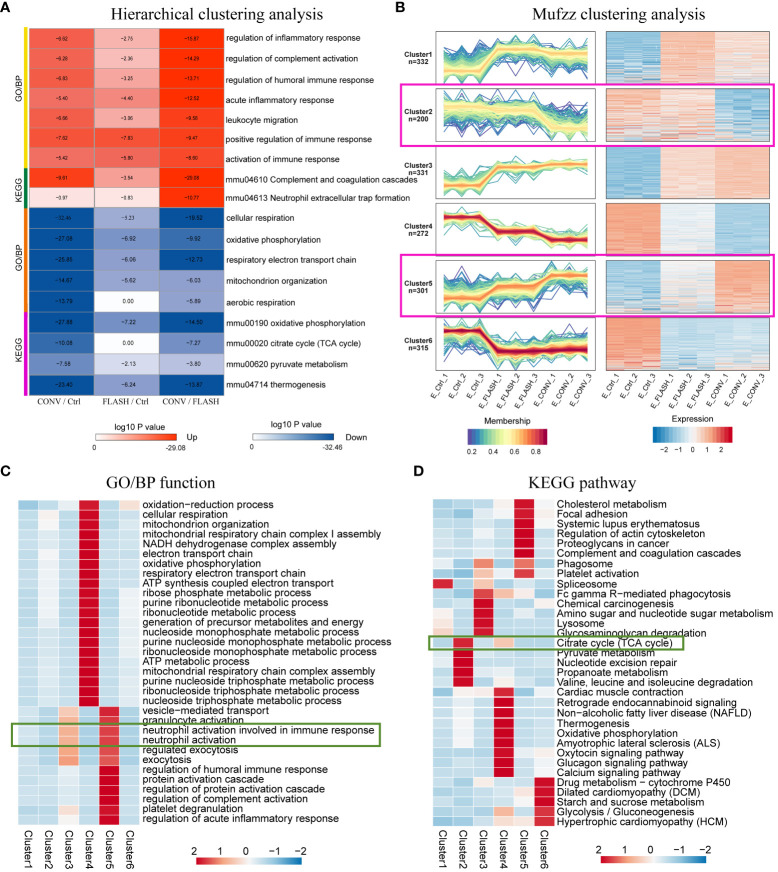
Expression pattern clustering of differential proteins. **(A)** The hierarchical clustering analysis presented as a heatmap. **(B)** The Mfuzz clustering analysis. The left column exhibits the protein expression levels, while the right column shows a heatmap showing expression levels. **(C)** GO/BP function enrichment heatmap. **(D)** KEGG pathway enrichment heatmap (*n* = 3 for each group).

To further elucidate the specific protein expression responses that resulted from FLASH irradiation, we applied GO and KEGG functional enrichment analysis for DEPs. Following this, hierarchical clustering was applied to identify relevant functions (GO/BP) or pathways (KEGG) from different comparison couples (CONV/Ctrl, FLASH/Ctrl, CONV/FLASH). The results were visualized as a heatmap (**Supplementary Figure S3**). By analyzing the functional differences displayed in the heatmap between the FLASH and CONV groups, we found that functions related to neutrophil-associated innate immune inflammation as well as tricarboxylic acid (TCA) cycle-related mitochondrial aerobic respiration were significantly enriched. The functions and pathways included the regulation of inflammatory response, regulation of complement activation, regulation of humoral immune response, acute inflammatory response, leukocyte migration, positive regulation of immune response, activation of immune response complement and coagulation cascades, neutrophil extracellular trap formation, cellular respiration, oxidative phosphorylation, mitochondrion organization, aerobic respiration, pyruvate metabolism, and citrate cycle (*p* < 0.001). These results were color-coded based on the magnitude of the log10 *p*-value and further presented in **Figure 3A**.

In addition to hierarchical clustering methods, we also employed a novel clustering approach called Mfuzz to analyze the protein expression patterns across the three groups, and proteins within the same cluster exhibit similar trends in expression changes (**Figure 3B**). From the clustering results, proteins in clusters 2, 4, and 5 exhibited the largest differences in abundance changes between the FLASH and CONV groups, while in clusters 1, 3, and 6, the abundance changes of proteins between the two irradiation modes were relatively consistent. After conducting enrichment analysis on proteins in different clusters in terms of GO/BP and KEGG, we found that proteins in clusters 2, 4, and 5, which exhibited the greatest differences, were predominantly associated with mitochondrial aerobic respiratory energy metabolism and neutrophil-involved immune responses (**Figures 3C, D**). This finding is consistent with the hierarchical clustering results, further elucidating and consolidating the distinct radiation response between the FLASH irradiation and CONV irradiation.

### FLASH irradiation impacts the expression of proteins related to the mitochondria and inflammation

**Figure 4:**
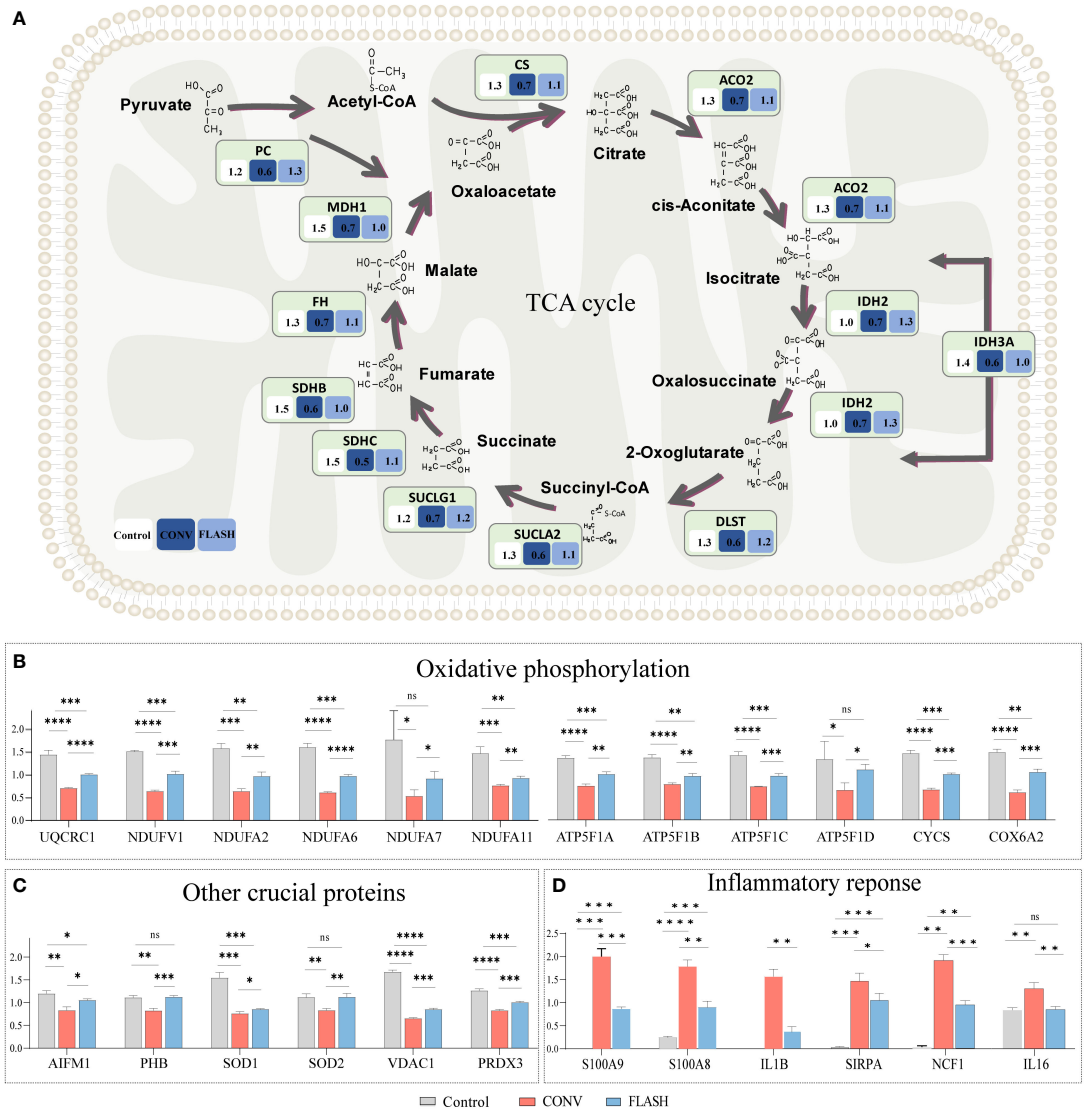
Quantification of pivotal proteins implicated in mitochondrial function and inflammatory response. **(A)** The quantification of proteins involved in the TCA cycle. The numbers in each box indicate the average relative quantitative value of proteins. **(B)** The quantification of proteins involved in oxidative phosphorylation pathways. **(C)** The quantification of proteins in other crucial functions of the mitochondria. **(D)** The quantification of proteins in inflammatory response (*n* = 3 for each group) *: *p* < 0.05, **: *p* < 0.01, ***: *p* < 0.001, ****: *p* < 0.0001. ns, nonsignificant.

Based on the aforementioned enrichment results of functions and pathways, we further analyzed the corresponding protein expression status in proteomics. The results demonstrated that CONV irradiation induced a significant reduction in the expression levels of key enzymes involved in the TCA cycle, such as citrate synthase (CS), aconitate hydratase (ACO2), isocitrate dehydrogenase (IDH2, IDH3A), dihydrolipoamide S-succinyl transferase (DLST), succinyl-CoA ligase (SUCLA2), succinate–CoA ligase (SUCLG1), succinate dehydrogenase (SDHB, SDHC), fumarate hydratase (FH), malate dehydrogenase (MDH1), and pyruvate carboxylase (PC). In contrast, the reduction magnitude regarding the expression of these key enzymes displayed a notable attenuation in the FLASH group (**Figure 4A**).

Similar alterations were observed in oxidative phosphorylation key proteins, among which the expressions of a series of proteins such as ubiquinol-cytochrome c reductase core protein 1 (UQCRC1), NADH: ubiquinone oxidoreductase subunits (NDUFV1, NDUFA2, NDUFA6, NDUFA7, NDUFA11), and ATP Synthase Subunit b (ATP5F1A, ATP5F1B, ATP5F1C, ATP5F1D) exhibited a relatively lower decline in magnitude in the FLASH group compared to the CONV group (**Figure 4B**; **Supplementary Figure S4**).

The expressions of other crucial proteins in the mitochondria, such as prohibitin (PHB), superoxide dismutase 1 (SOD1), and voltage-dependent anion channel 1 (VDAC1), were also investigated. PHB plays a critical role in mitochondrial proliferation (30). SOD1 is an antioxidant enzyme protecting the cell from reactive oxygen species (ROS) toxicity (31). VDAC1 is the gatekeeper for the passage of metabolites, nucleotides, and ions (32). The expressions of these crucial proteins also exhibited a similar trend in the FLASH and CONV groups (**Figure 4C**). The inflammatory proteins, including S100A8, S100A9, IL1B, SIRPA, NCF1, and IL16, were upregulated in both the CONV and FLASH groups, while the increase in magnitude regarding the expressions of these proteins in the FLASH group was significantly lower than that in the CONV group (**Figure 4D**).

### TEM and PRM validation of proteomic findings

**Figure 5:**
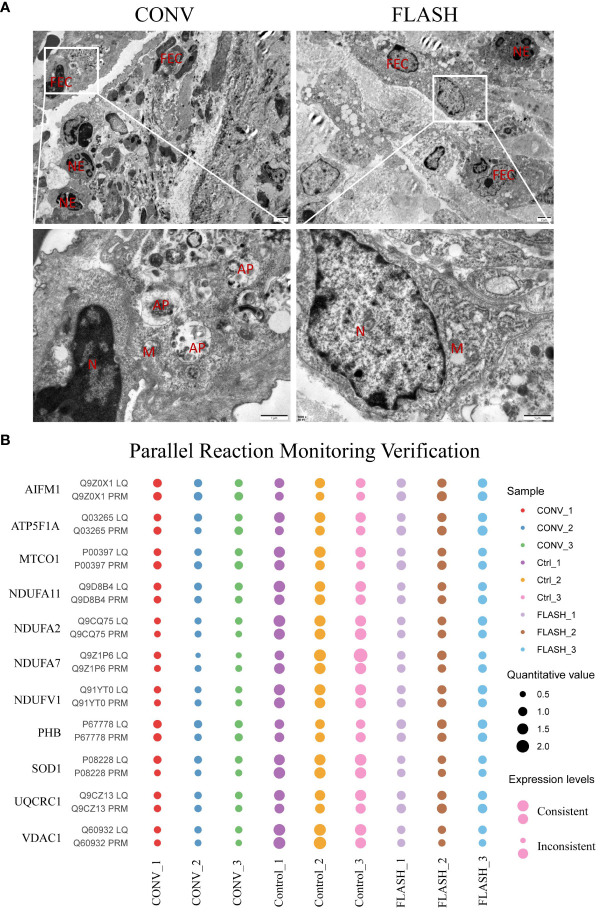
The results of transmission electron microscopy (TEM) and parallel reaction monitoring. **(A)** The representative images of TEM. FEC, flat epithelial cell; NE, neutrophil; AP, autophagosome; N, nucleus; M, mitochondria. **(B)** The PRM results of some typical mitochondrial proteins. LQ, label-free quantitative proteomics; PRM, parallel reaction monitoring (*n* = 3 for each group).

To validate the proteomic discoveries, we conducted TEM experiments to observe changes in tissue and cellular morphology, as well as PRM for the quantification of mitochondrial-targeted proteins. In the CONV group, TEM imaging revealed that the mitochondria exhibited swelling, blurred structure, disappearance or fragmentation of cristae, decreased electron density of the matrix, and vacuolization. In contrast, mitochondrial swelling and blurred structure were not obvious in the FLASH group. Additionally, in the CONV group, a higher number of infiltrated neutrophils and autophagic vacuoles were observed among epithelial cells, whereas in the FLASH group, a lower number of infiltrated neutrophils was noted (**Figure 5A**).

The PRM technology is a highly precise mass spectrometry that enables the accurate measurement of target proteins/peptides through selective detection. In this study, 12 target proteins including AIFM1, ATP5F1A, MTCO1, NDUFV1, NDUFA2, PHB, SOD1, and so on were performed for PRM quantification. Among them, the expression levels of 11 proteins in the three groups were consistent with the 4D label-free results, which reflected the reproducibility of the proteins (**Figure 5B**).

## Discussion

**Figure 6:**
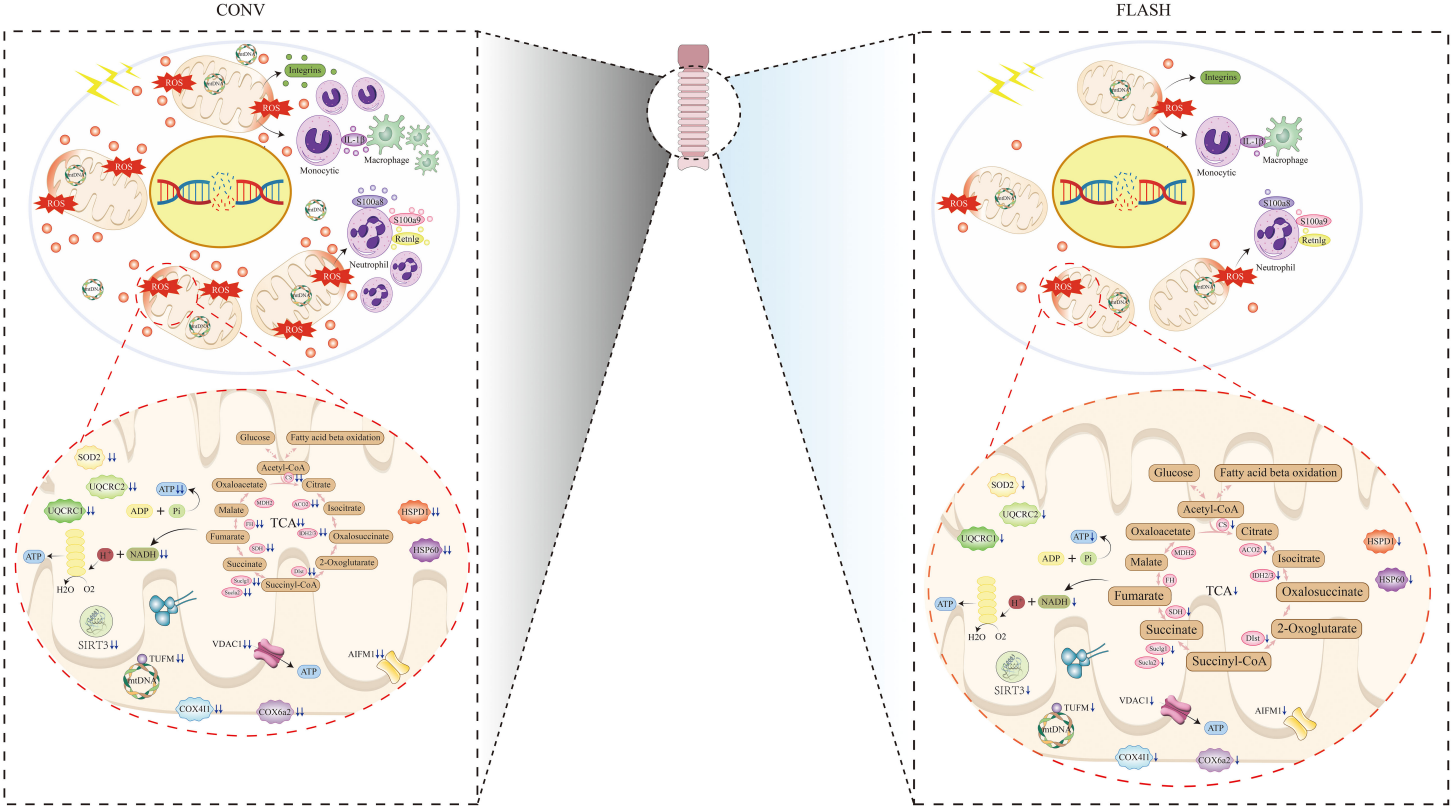
Schematic illustration of CONV irradiation compared with FLASH irradiation on esophageal tissue.

In this study, we focus on the sparing effect of FLASH irradiation on normal esophageal tissue, both in histological and protein expression levels. A total of three groups, namely, the control, CONV, and FLASH groups, were set up. The sparing effect of FLASH irradiation was confirmed by comparing the histological damage status of esophageal tissue between the FLASH and CONV groups. Then, the study further conducted a systematic analysis and verification of the disparities in protein expression between the FLASH and CONV groups, aiming to elucidate the molecular responses associated with the sparing effect of FLASH irradiation on normal esophageal tissue. These results demonstrated that FLASH irradiation was associated with alleviated damage of proteins responsible for mitochondrial functions, including the TCA cycle and oxidative phosphorylation. Furthermore, a reduction in acute inflammatory responses was also observed in the FLASH group. The schematic illustration is depicted in **Figure 6**.

The sparing effect of FLASH irradiation is of solid significance during clinical practice for the treatment of esophageal cancer using radiotherapy. Upon diagnosis of esophageal cancer, approximately 70% to 80% of esophageal cancer patients were involved with invasion of adjacent tissues or distant metastasis, posing challenges and risks to surgical intervention (33). For cases where chemotherapy and existing targeted drugs cannot provide effective therapeutic performance, RT plays a pivotal role in the treatment of esophageal carcinoma. However, it is imperative to acknowledge that RT also entails the risk of radiation-related side effects, potentially impacting the prescribing dosage choice. Esophagitis is a common radiation-induced complication which emerges approximately 2 to 3 weeks during radiotherapy. Patients with esophagitis may experience dysphagia accompanied by an exacerbation in the sensation of food obstruction and a burning sensation or discomfort behind the sternum. In severe cases, dehydration, malnutrition, electrolyte imbalance, or weight loss may ensue. A small proportion of severely affected cases may exhibit symptoms such as esophageal hemorrhage and perforation, which would affect the complete implementation of radiotherapy. FLASH RT holds promise in mitigating the toxicity to adjacent non-malignant tissues, which can potentially reduce the risk of complications such as esophagitis and ensure the complete implementation of radiotherapy. It is of clinical significance to further explore the protein expression responses in the sparing effect, aiming to promote the integration of FLASH into clinical application.

Previous research on radiation injuries primarily relied on proteomics based on two-dimensional gel electrophoresis (2DE) (34, 35). This traditional approach is not conducive to a comprehensive observation of a large number of protein expression level changes. In this study, we employed a novel protein quantification technique that does not require staining or radiolabeling prior to electrophoresis in 2DE. Through the analysis of protein-digested peptide segments using liquid chromatography-mass spectrometry, the signal intensities of the corresponding peptide segments in different samples can be compared, and the relative quantification of the corresponding proteins was facilitated, enabling a comprehensive analysis of protein level alterations that resulted from FLASH or CONV irradiation. Finally, alleviated acute inflammation and mitochondrial damage were observed in the FLASH group when compared with the CONV group.

Published studies consistently emphasize mitochondrial proteins as the most radiation-sensitive protein class (36, 37). In our study, CONV irradiation significantly impacts mitochondrial-related functions such as the TCA cycle and oxidative phosphorylation, leading to a notable downregulation of corresponding proteins. This observation aligns with previous research on radiation effects where gas chromatography/mass spectrometry metabolomics was employed to investigate the response to ionizing radiation (38). The study mentioned above also found a decrease in the intermediate metabolites of the TCA cycle. Interestingly, the proteomic analysis in our study revealed that the key enzymes involved in the TCA cycle were also downregulated after CONV irradiation, which potentially reminds a causal relationship with the decreased levels of intermediate metabolites. Though the mitochondrial-function-related protein levels were also affected by FLASH irradiation, the downregulation extents of these proteins were significantly alleviated. This finding is consistent with previous studies on FLASH-induced mitochondrial damage (39, 40). Different from existing studies, our study utilized whole proteomic analysis to facilitate a better observation of functional clusters using global protein analysis, thereby displaying a direct comparison on protein levels of different pathways between the FLASH and CONV groups. According to our findings, we deduced that FLASH irradiation may minimize disruption to cellular homeostasis by attenuating the impact on mitochondrial functionality. Furthermore, the preservation of mitochondrial functionality may also promote cell repair capacity against radiation damage, as demonstrated by existing studies (41, 42). Further validation of these findings was needed in the following studies.

It was reported that CONV irradiation would typically elicit a robust inflammatory response. Following exposure to radiation, the radiation-induced damage molecular patterns (DAMPs) would trigger the release of inflammatory mediators, attract cytokines and chemokines to the damaged site, and induce proinflammatory responses. In addition to the direct inflammatory response induced by CONV irradiation, mitochondrial damage can further aggravate the inflammatory response (43). In previous studies regarding skin toxicity after FLASH irradiation, decreased levels of proinflammatory mediators such as TNF-α and IL-6 were observed. These results were further supported by our findings that a significant attenuation of the inflammatory response was displayed in the FLASH group (25, 44). However, in contrast to previous experiments that relied on specific individual cytokines to differentiate various types of inflammatory responses between the CONV and FLASH groups, we employed proteomics data to elucidate these distinctions and clearly delineated the most significant alterations along the entire pathway of inflammatory proteins.

It is worth noting that the size of the radiation field plays a crucial role and deserves emphasis in our study. According to our design, the radiation field encompasses not only the esophageal tissue but also extends throughout the entire thoracic cavity, thereby exposing vital organs such as the lungs, esophagus, and heart to radiation. This comprehensive design facilitates concurrent research on multiple chest organs. In fact, we are currently compiling and analyzing data regarding changes in lung tissue following exposure to FLASH radiation, which will be presented in subsequent studies.

This study has several limitations. Firstly, the proteome experimental data are extensive, and there is a substantial number of differentially expressed proteins between the two radiation modalities. However, this study specifically focuses on researching and discussing the most significant mitochondrial functions and inflammatory responses. Secondly, we only acquired proteomic data based on mass spectrometry while lacking information on the post-translational modification, transcriptome, and copy number variations. This limitation confines our study to proteomics, and future research will employ multi-omics data to further elucidate the mechanism of FLASH.

## Conclusions

In summary, FLASH irradiation displayed a sparing effect on esophageal tissue when compared with CONV irradiation. The mitigation of mitochondrial damage and acute inflammation may serve as the key elements contributing to the sparing effects of FLASH irradiation on the esophagus. Moving forward, further in-depth research is essential to explore the underlying mechanisms of these sparing effects.

## Data availability statement

The datasets presented in this study can be found in online repositories. The names of the repository/repositories and accession number(s) can be found in the article/**Supplementary Material**.

## Ethics statement

The animal study was approved by the Experimental Animal Ethics Committee of Cancer Hospital, Chinese Academy of Medical Sciences. The study was conducted in accordance with local legislation and institutional requirements.

## Author contributions

WR: Conceptualization, Data curation, Formal analysis, Investigation, Methodology, Visualization, Writing – original draft, Writing – review & editing. LH: Data curation, Formal analysis, Investigation, Methodology, Writing – review & editing. KZ: Data curation, Methodology, Writing – review & editing. HC: Data curation, Formal analysis, Investigation, Methodology, Writing – review & editing. XF: Data curation, Formal analysis, Investigation, Methodology, Writing – review & editing. ZJ: Data curation, Formal analysis, Investigation, Methodology, Writing – review & editing. FS: Data curation, Formal analysis, Investigation, Methodology, Writing – review & editing. JD: Conceptualization, Writing – review & editing. YG: Conceptualization, Funding acquisition, Project administration, Writing – review & editing. JH: Conceptualization, Funding acquisition, Project administration, Supervision, Writing – review & editing.
