# Supplementary material for: The sparing effect of ultra-high dose rate irradiation on the esophagus
Source: Front Oncol. 2024 Jul 12;14:1442627. doi: 10.3389/fonc.2024.1442627 (PMC11272628; doi:10.3389/fonc.2024.1442627)
Supplement: Supplementary file 1 [file DataSheet_1.docx]

**Table S1. Irradiation parameters**.

| Delivery mode | Prescribed dose (Gy) | Beam parameters | | | | | | |
| --- | --- | --- | --- | --- | --- | --- | --- | --- |
|  |  | Beam energy (MeV) | Pulse repetition frequency (Hz) | Pulse width (μs) | Number of pulses | Treatment time (s) | Mean dose rate (Gy/s) | Instantaneous dose rate (Gy/s) |
| CONV | 20 | 10 | 200 | 3.3 | / | 200 | 0.1 | / |
| FLASH | 20 | 10 | 6.25 | 3.3 | 2 | 0.16 | 125 | 3.03×10^6^ |

CONV: conventional dose rate radiotherapy.

FLASH: ultra-high-dose-rate radiotherapy.


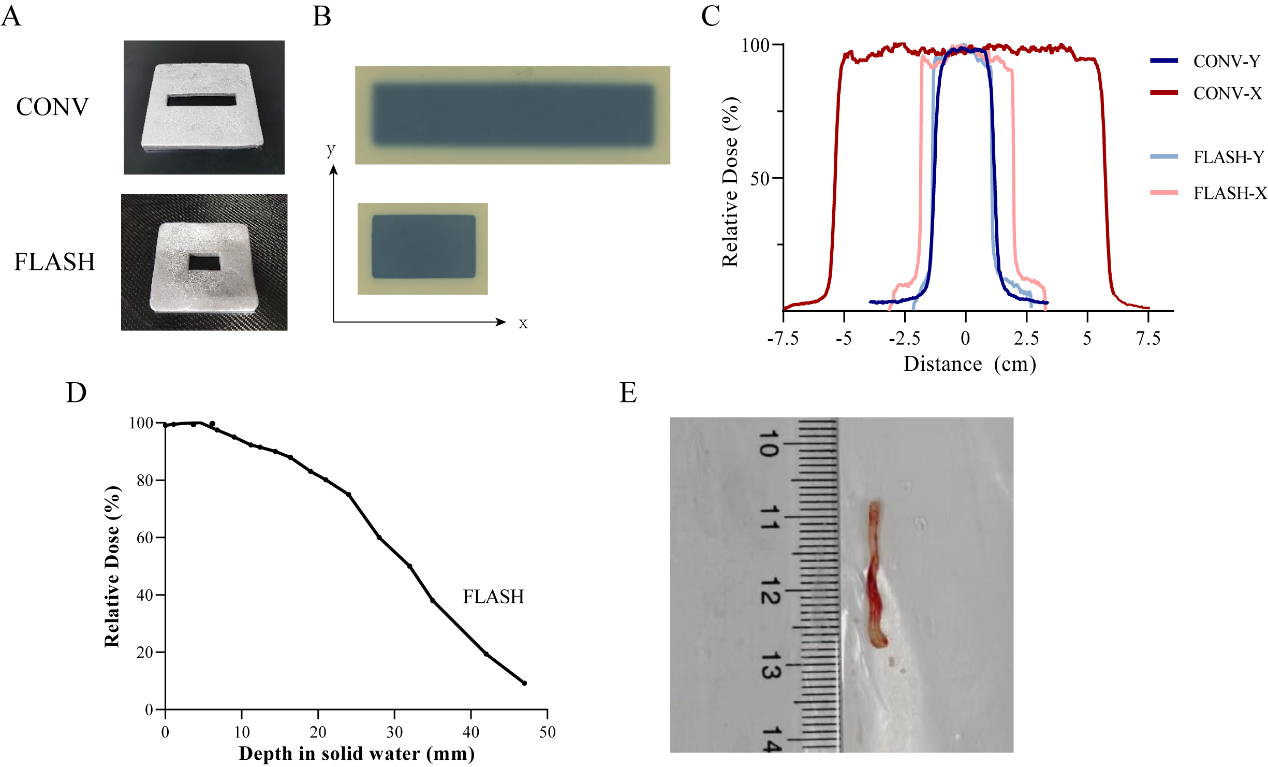


Figure S1. A The custom-built lead blocks (1 cm thickness) for FLASH and CONV irradiation. A 2.5 cm × 3.5 cm hollow in the middle allows for the irradiation of one mouse at a time in the FLASH mode, while a 2.5 cm × 10 cm hollow in the middle accommodates the irradiation of three mice at a time in the CONV mode. B The dose films for the CONV and FLASH irradiation modes, which were measured in a water-equivalent polystyrene phantom. C Transverse dose profile of FLASH vs. CONV irradiation. D The Depth dose profile of FLASH irradiation. E An anatomical photograph of a mouse esophagus specimen.


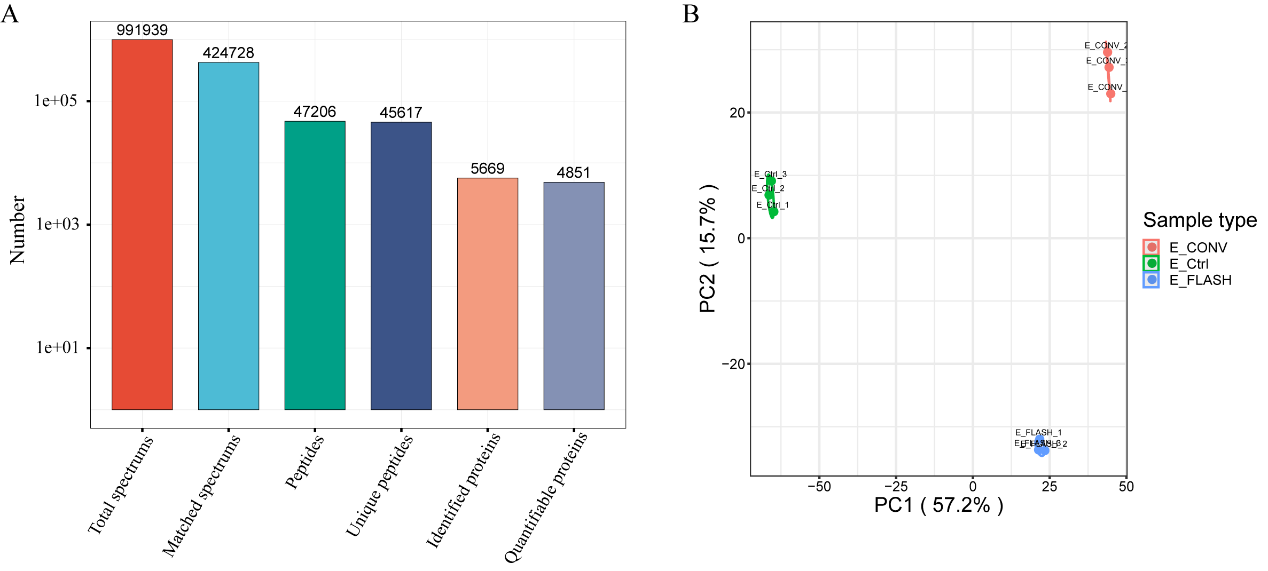


Figure S2. A A comprehensive overview of quantitative proteomics data. B Pearson’s correlation coefficient (PCC) analysis among all samples.


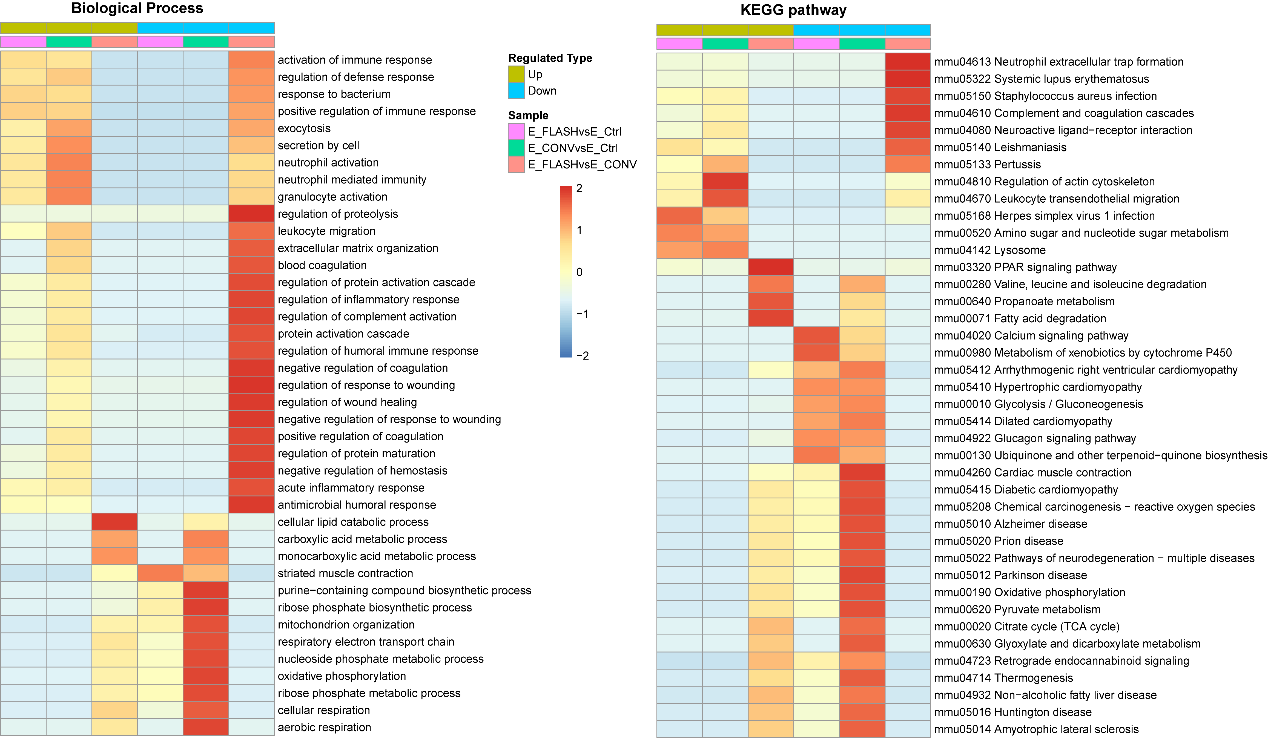


Figure S3. The hierarchical clustering analysis of biological process (BP) function and KEGG pathway.


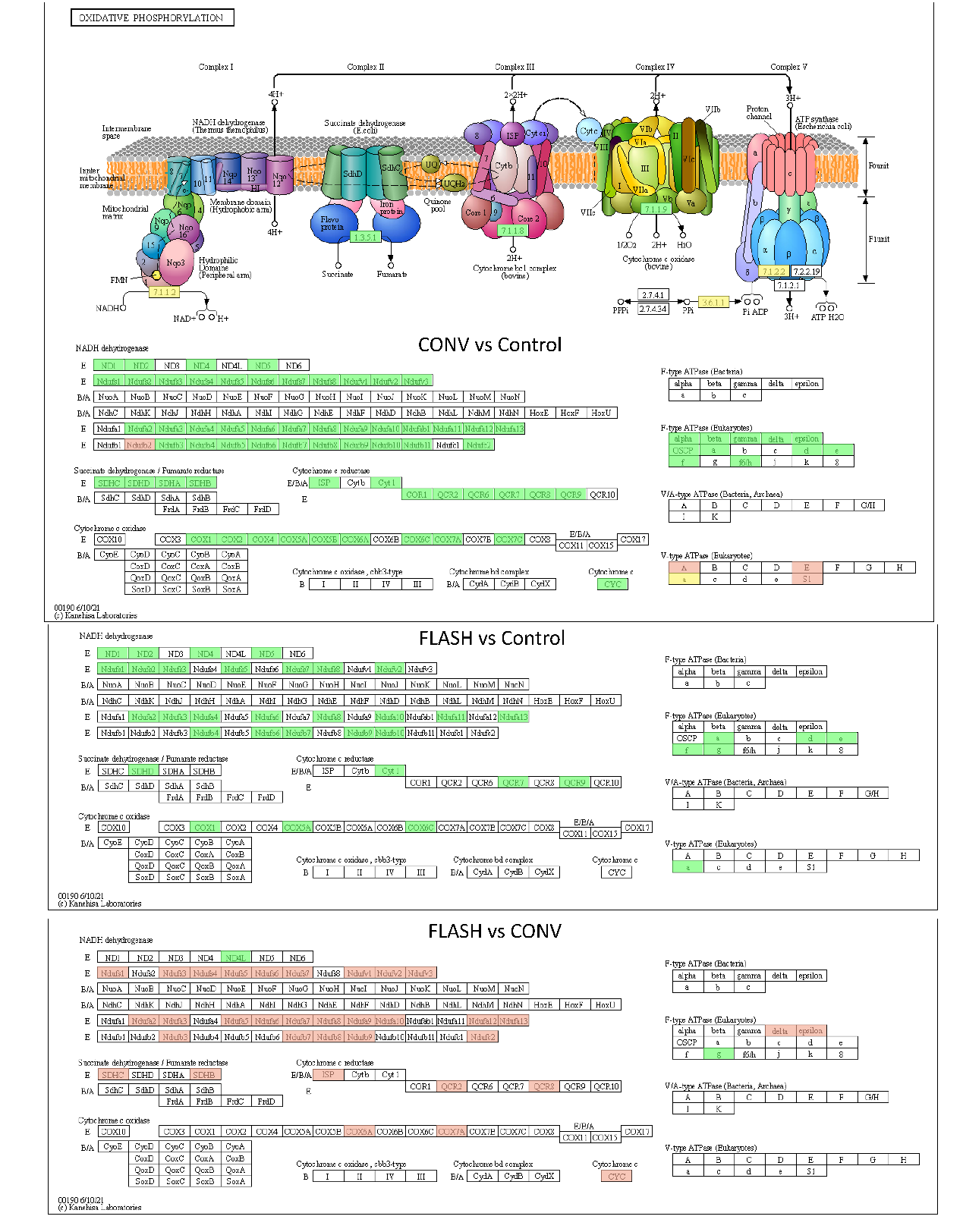


Figure S4. Differential enrichment of proteins in the oxidative phosphorylation pathway among various comparison groups. Green indicates a relative decrease in protein expression levels in the preceding group compared to the subsequent group, while red signifies an increase.
